# 52350 PKM2 mediates anti-tumor immunity and T cell dysfunction

**DOI:** 10.1017/cts.2021.630

**Published:** 2021-03-31

**Authors:** Geoffrey Markowitz, Yi Ban, Michael Crowley, Diamile Tavarez, Stephen T.C. Wong, Kenneth Chang, Andrea Schietinger, Nasser Altorki, Vivek Mittal

**Affiliations:** 1 Weill Cornell Medicine; 2 Houston Methodist; 3 Cold Spring Harbor Laboratories; 4 Memorial Sloan Kettering Cancer Center

## Abstract

ABSTRACT IMPACT: T cell dysfunction is a dominant suppressor of anti-tumor immunity, reducing immunotherapeutic efficacy and benefit to patients; our work will identify novel mediators of this process for both therapeutic potential and underlying mechanism, allowing for both potential immediate clinical utility and identification of future targets based on new mechanistic insights. OBJECTIVES/GOALS: T cell dysfunction is a dominant suppressor of anti-tumor immunity, reducing immunotherapeutic efficacy and clinical benefit to the majority of patients. We aim to interrogate a novel mediator of dysfunction identified from transcriptome analyses, pyruvate kinase muscle isozyme isoform 2 (PKM2), for therapeutic utility and underlying mechanism. METHODS/STUDY POPULATION: Transcriptome analyses of CD8+ lymphocytes from tumor-bearing lungs from both murine KrasG12D p53-/- and human non-small cell lung cancer (NSCLC) patients were performed, and differentially expressed genes identified. Flow cytometric analyses for PKM isoform expression and effects of target knockdown on accumulation of dysfunctional characteristics, including checkpoint and transcription factor expression, proliferation, and cytokine production, were performed using an in vitro co-culture of murine antigen-specific T (OT-I) cells and antigen-expressing NSCLC (HKP1-ova) cells. In vivo examination of the same was performed using adoptive transfer of OT-I cells into immunocompetent recipient mice with engraftment of HKP1-ova cells, and subsequent evaluation of mouse survival and T cell phenotypes. RESULTS/ANTICIPATED RESULTS: Transcriptome analyses demonstrated that PKM expression was upregulated in dysfunctional T cells from both murine and human samples. This was confirmed both in vitro with co-culture and in vivo with adoptive transfer approaches, with both activated and dysfunctional OT-I cells expressing higher levels of isoform 2 of PKM than naive OT-I cells. Expression of PKM2 mimicked the kinetics of the transcription factor Tox, a known driver of dysfunction, and knockdown of PKM2 resulted in reduced granzyme B expression, and increased proportions of progenitors with fewer terminally differentiated dysfunctional cells. Knockdown of PKM2 in adoptively-transferred OT-I cells led to enhanced tumor control; results are being extended to other tumor models, and T cells metabolically profiled with PKM2 manipulation. DISCUSSION/SIGNIFICANCE OF FINDINGS: This work identified a novel mediator of dysfunction whose targeting has the potential to enhance anti-tumor immunity. Mechanistically, targeting PKM2 led to altered T cell differentiation to a dysfunctional state, linking metabolic phenotypes to these traits and underlining the importance and therapeutic potential of T cell metabolic pathways.

